# Clinical Test Performance of a Rapid Point-of-Care Syphilis Treponemal Antibody Test: A Systematic Review and Meta-analysis

**DOI:** 10.1093/cid/ciaa350

**Published:** 2020-06-24

**Authors:** Claire C Bristow, Jeffrey D Klausner, Anthony Tran

**Affiliations:** 1 Division of Infectious Diseases and Global Public Health, University of California, San Diego, San Diego, California, USA; 2 Department of Medicine, University of California, Los Angeles, Los Angeles, California, USA; 3 District of Columbia Department of Forensic Sciences, Public Health Laboratory Division, Washington, DC, USA

**Keywords:** diagnosis, syphilis, rapid test, Treponema, point-of-care

## Abstract

We reviewed relevant syphilis diagnostic literature and conducted a meta-analysis to address the question, “What is the sensitivity and specificity of the Syphilis Health Check, a rapid qualitative test for the detection of human antibodies to *Treponema pallidum*.” The Syphilis Health Check is the only rapid syphilis test currently cleared by the Food and Drug Administration (FDA). We conducted a systematic review and a meta-analysis using Bayesian bivariate random-effects and fixed-effect models to create pooled estimates of sensitivity and specificity of the Syphilis Health Check. We identified 5 test evaluations published in the literature and 10 studies submitted to the FDA and for a Clinical Laboratory Improvement Amendments waiver application. The pooled sensitivity (95% CI) from the laboratory evaluations (n = 5) was 98.5% (92.1–100%), while pooled specificity was 95.9% (81.5–100.0%). The pooled sensitivity for prospective studies (n = 10) was 87.7% ( 71.8–97.2%), while pooled specificity was 96.7% (91.9–99.2%). Using nontreponemal supplemental testing, the sensitivity improved to a pooled sensitivity of 97.0% (94.8–98.6%). The Syphilis Health Check may provide accurate detection of treponemal antibody.

Syphilis, caused by infection with the bacterium *Treponema pallidum*, is a re-emerging public health problem that can lead to significant complications if left untreated. In addition, syphilis has been demonstrated to facilitate human immunodeficiency virus acquisition and transmission [1–3]. In the United States, syphilis rates have increased nearly every year since 2000. In 2017, the rate was 9.5 per 100 000, the highest rate since 1993 [4]. While men who have sex with men account for the majority of cases reported to the Centers for Disease Control and Prevention, the increases in syphilis incidence among women (up 155.6% between 2013 and 2017 [4]) are of significant concern due to the impact that syphilis can have on pregnant women and their fetuses.

Routine screening and early treatment are essential to reduce the burden and impact of this disease. Fortunately, syphilis is curable; generally, penicillin G is utilized to treat persons in all stages of syphilis [5]. Congenital syphilis can be prevented by screening early in pregnancy, treating syphilis-infected pregnant women, and preventing reinfection [6, 7].

Globally, over the past 15 years, rapid tests that can be used at the point-of-care have been developed that detect treponemal antibody using fingerstick whole-blood specimens. In global settings, rapid syphilis tests play an important role in prompt diagnosis [8]. These tests can be performed without laboratory processing, at the point-of-care and while the patient waits, thus reducing the potential for loss to follow-up [9–13]. With a shortened time to diagnosis, patients may be treated much more quickly, resulting in reduced complications from untreated infection as well as reducing the spread of syphilis to others. Most rapid tests that are available globally detect treponemal antibody and therefore cannot be used to determine if the infection is current and requires treatment. Testing with a nontreponemal test (anticardiolipin antibody test) is therefore still recommended in some settings to inform patient management [14, 15]. However, a nonreactive treponemal test may be useful to rapidly rule out syphilis.

The Syphilis Health Check (Diagnostics Direct, LLC, Stone Harbor, NJ) is a rapid qualitative test for detection of human antibodies to *T. pallidum* in serum, plasma, or whole blood. The test utilizes antihuman immunoglobulins gold conjugate and highly purified *T. pallidum* recombinant proteins for specific detection of anti–*T. pallidum* antibodies [16]. That test detects both immunoglobulin (Ig) G and IgM using TP-15, TP-17, and TP-44 recombinant syphilis antigens. The test works by lateral flow of the sample through the absorbent device where, in the presence of *T. pallidum* antibodies, the antihuman immunoglobulins/protein dye conjugate binds to the human immunoglobulins forming an antigen–antibody complex. That complex produces a pink-colored band to indicate a reactive result. In the absence of anti–*T. pallidum* antibodies, no line is present in the reaction zone to indicate a nonreactive result. A reagent control mechanism is included in the test in which unbound conjugate binds to the reagents in the control zone producing a pink-colored band. This band must be present for the test to be valid.

To review the performance of the Syphilis Health Check we conducted a meta-analysis that combines data from all available evaluations of the Syphilis Health Check. We created pooled estimates of sensitivity and specificity for the rapid test.

## METHODS

We searched Medline, Embase, CINAHL, Scopus, and the Cochrane Library databases using search terms that included the following: (Syphilis OR *Treponema pallidum*) AND (Syphilis Health Check OR Rapid test OR point-of-care test OR point of care test OR POC test OR rapid point-of-care test OR rapid point of care test OR RPOC test OR diagnostic test OR combination test OR dual test OR multiplex test OR ASSURED OR rapid syphilis test OR RST OR saliva test OR immunochromatographic test OR finger-stick test). Abstracts were reviewed for relevance. We excluded articles that were not in English; were duplicates; had no authors; had no abstracts; were opinion, case reports, or reviews; and were not related to syphilis or point-of-care tests. Studies and data were included that evaluated the Syphilis Health Check rapid test. In addition to those studies identified in the literature, we included the studies from the manufacturer’s Food and Drug Administration (FDA) application [17] for clearance of the Syphilis Health Check test as well as the Clinical Laboratory Improvement Amendments (CLIA) waiver application study data that were described in the package insert (REV P 02/17) [16]. The FDA studies included retrospective studies using stored or purchased serum specimens. The prospective FDA and CLIA studies used prospectively collected clinical specimens.

For the meta-analysis of diagnostic accuracy, the data extracted included study title, authors, year of publication, reference test type, specimen type, and study location. We calculated sensitivity and specificity for each study individually and used the exact binomial method to calculate 95% confidence intervals (CIs).

We used Bayesian bivariate random-effects and fixed-effect models meta-analysis to calculate pooled sensitivity and specificity of the Syphilis Health Check test using SAS PROC MCMC (SAS Institute, Cary, NC) [18]. We considered the fixed-effect model to be a better fit if the deviance information criterion (DIC) was less than 10 larger than the DIC of the random-effects model [ie, (DIC_FIXED_—DIC_RANDOM_) <10]; otherwise, the random-effects model was used to calculate pooled estimates of sensitivity and specificity [19]. SAS version 9.4 was used for all other data analyses.

## RESULTS

We identified 5 test evaluations published in the literature as well as studies submitted to the FDA and for the CLIA waiver application that we included in our meta-analysis. All studies (n = 15) [17, 20–24] used treponemal tests as the reference tests, while some included nontreponemal test result information. We separated performance results by study type, laboratory-based retrospective versus prospective. There were 10 prospective studies identified—4 from the literature describing clinic-based prospective evaluations, 1 from the CLIA application, and 5 from the FDA application. Among the prospective studies, some studies used whole-blood fingerstick specimens and some used sera (Table 1).

**Table 1.** Studies of Syphilis Health Check Included in Meta-analysis, Reference Tests, Specimen Types, and Study Locations

Author, Year, or Study Name From Regulatory Evaluation	Reference Tests	Specimen Type	Study Location
Nakku-Joloba et al, 2016 [21]	RPR−/TPHA− = negative; RPR+ /rapid treponemal test+ = positive	Blood, not specified	Uganda
Matthias et al, 2016 [20]	Trep-Sure EIA/RPR	Fingerstick, whole blood	Florida, USA
Fakile et al, 2019 [23]	Trep-Sure EIA/RPR	Fingerstick, whole blood	North Carolina, USA
Fakile et al, 2019 [24]	TPPA/RPR	Fingerstick, whole blood	Michigan, USA
CLIA study (package insert) [16]	CIA/RPR (TPPA for tiebreaker)	Fingerstick, whole blood	3 sites across USA
FDA university clinic site [17]	FTA-Abs	Serum	USA
FDA hospital clinic site [17]	TPHA	Serum	USA
FDA study site 1 [17]	TPPA	Serum	USA
FDA study site 2 [17]	TPPA	Serum	USA
FDA study site 3 [17]	EIA	Serum	USA
FDA retrospective, known positive [17]	TPPA	Serum	USA
FDA retrospective, pregnant women [17]	TPPA	Serum	USA
FDA suspected positive [17]	TPHA	Serum	USA
FDA clinically diagnosed [17]	TPPA and FTA-Abs	Serum	USA
Pereira et al, 2018 [22]	TPPA/CIA/EIA and RPR	Serum	USA

Abbreviations: CIA, chemiluminescence immunoassay; EIA, enzyme immunoassay; FDA, Food and Drug Administration; FTA-Abs, fluorescent treponemal antibody absorption test; RPR, rapid plasma reagin; TPHA, *Treponema pallidum* hemagglutination assay; TPPA, *Treponema pallidum* particle agglutination assay

The sensitivity and specificity estimates for each study are included in Tables 2–4. The sensitivity and specificity estimates from the prospective studies (n = 10) ranged from 50.0% to 100% and 50% to 100%, respectively. For laboratory-based evaluations on stored serum specimens (n = 5), the sensitivity and specificity estimates ranged from 88.7% to 100% and 83.3% to 100%, respectively.

**Table 2.** Meta-analysis of Prospective Evaluations of the Syphilis Health Check Rapid Test Using Treponemal Tests as Reference Tests

		Treponemal Ref Test					Estimate (95% CI)		
Author, Year, or Study Name From Regulatory Evaluation	Specimen Type	TP	TN	FP	FN	N	Sensitivity	Specificity
Clinical studies								
Nakku-Joloba et al, 2016	Blood (not specified)	88	108	9	10	215	89.8% (82.0–95.0%)	92.3% (85.9–96.4%)
Matthias et al, 2016	Fingerstick,whole blood	10	172	16	4	202	71.4% (41.9–91.6%)	91.5% (86.6–95.1%)
Fakile et al, 2019	Fingerstick, whole blood	8	523	23	8	562	50.0% (24.7–75.4%)	95.8% (93.8–97.3%)
Fakile, Markowitz et al, 2019	Fingerstick, whole blood	13	930	6	13	962	50.0% (29.9–70.1%)	99.4% (98.6–99.8%)
Regulatory studies								
CLIA study (package insert)^a^	Fingerstick, whole blood	197	208	6	4	415	98.0% (95.0–99.5%)	97.2% (94.0–99.0%)
FDA university clinic site	Serum	27	6	6	0	39	100.0% (87.2–100.0%)	50.0% (21.1–78.9%)
FDA hospital clinic site	Serum	6	44	0	0	50	100.0% (54.1–100.0%)	100.0% (92.0–100.0%)
FDA study site 1	Serum	21	365	8	6	400	77.8% (57.7–91.4%)	97.9% (95.8–99.1%)
FDA study site 2	Serum	4	85	0	0	89	100.0% (39.8–100.0%)	100.0% (95.8–100.0%)
FDA study site 3	Serum	9	193	2	1	205	90.0% (55.5–99.8%)	99.0% (96.3–99.9%)
Total	…	383	2634	76	46	3139	…	…
Pooled prospective (random-effects model)	…	…	…	…	…	…	87.7% (71.8–97.2%)	96.7% (91.9–99.2%)

DIC_fixed_–DIC_random_ = 221.076–92.697.

Abbreviations: CI, confidence interval; CLIA, Clinical Laboratory Improvement Amendments; DIC, deviance information criterion; FDA, Food and Drug Administration; FN, false negative; FP, false positive; N, sample size; Ref, reference; TN, true negative; TP, true positive.

^a^CLIA study used nontreponemal results in addition to the treponemal results in the patient infected status determination.

**Table 3.** Meta-analysis of Laboratory Evaluations of the Syphilis Health Check Rapid Test Using Treponemal Tests as Reference Tests

		Treponemal Ref Test					Estimate (95% CI)	
Study Name From Regulatory Evaluation or Author and Year	Specimen Type	TP	TN	FP	FN	N	Sensitivity	Specificity
FDA retrospective, known positive	Serum	290	20	4	1	315	99.7% (98.1–100.0%)	83.3% (62.6–95.3%)
FDA retrospective, pregnant women	Serum	94	68	0	0	162	100.0% (96.2–100.0%)	100.0% (94.7–100.0%)
FDA suspected positive	Serum	87	10	0	0	97	100.0% (95.9–100.0%)	100.0% (69.2–100.0%)
FDA clinically diagnosed	Serum	164	0	0	0	164	100.0% (97.8–100.0%)	NA
Pereira et al, 2018	Serum	669	607	45	85	1406	88.7% (86.3–90.9%)	93.1% (90.9–94.9%)
Total	…	1304	705	49	86	2144	…	…
Pooled retrospective (random effects model)	…	…	…	…	…	…	98.5% (92.1–100.0%)	95.9% (81.5–100.0%)

Abbreviations: CI, confidence interval; FDA, Food and Drug Administration; FN, false negative; FP, false positive; N, sample size; NA, not applicable; Ref, reference; TN, true negative; TP, true positive.

**Table 4.** Meta-analysis of Evaluations of the Syphilis Health Check Rapid Test That Use Both Treponemal and Nontreponemal Tests as Reference Tests

		Trep and Nontrep Ref Tests					Estimate (95% CI)	
Author, Year	Specimen Type	TP	TN	FP	FN	N	Sensitivity	Specificity
Clinical studies								
Nakku-Joloba et al, 2016	Blood (not specified)	81	103	2	4	190	95.3% (88.4–98.7%)	98.1% (93.3–99.8%)
Matthias et al, 2016	Fingerstick, whole blood	6	171	16	1	194	85.7% (42.1–99.6%)	91.4% (86.5–95.0%)
Fakile et al, 2019	Fingerstick, whole blood	7	531	24	0	562	100.0% (59.0–100.0%)	95.7% (93.6–97.2%)
Regulatory study								
CLIA study (package insert REV. P 02/17)	Fingerstick, whole blood	197	208	6	4	415	98.0% (95.0–99.5%)	97.2% (94.0–99.0%)
Total	…	291	1013	48	9	1361	…	…
Pooled prospective (fixed effect model)	…	…	…	…	…	…	97.0% (94.8–98.6%)	95.5% (94.2–96.7%)

DIC_fixed_–DIC_random_ = 41.142–37.137. Nakku-Joloba et al and Matthias et al: Reference test positive required a RPR and treponemal reference reactive result; reference test negative was defined as those both RPR and treponemal nonreactive. Fakile et al: Reference test positive required a RPR and treponemal reference reactive result; reference test negative was defined as those RPR and treponemal reference test nonreactive, RPR reactive and treponemal reference as nonreactive, or RPR nonreactive and treponemal reference test reactive. The CLIA waiver study used the reverse algorithm with a chemiluminescent immunoassay (CIA) as the primary treponemal test and RPR as the supplemental nontreponemal test. If the RPR was nonreactive, the TPPA was used as the secondary treponemal test.

Abbreviations: CI, confidence interval; CLIA, Clinical Laboratory Improvement Amendments; DIC, deviance information criterion; FN, false negative; FP, false positive; N, sample size; Nontrep, nontreponemal; Ref, reference; RPR, rapid plasma reagin; TN, true negative; TP, true positive; TPPA, *Treponema pallidum* particle agglutination; Trep, treponemal.

The pooled sensitivity for prospective studies was 87.7% (95% CI, 71.8–97.2%) and the pooled specificity was 96.7% (95% CI, 91.9–99.2%) (Table 2). For the 4 prospective studies identified in the literature, the sensitivity was lower than that in all of the 5 FDA studies and with wide CIs. We pooled the results from those 4 prospective studies identified from the literature in a random-effects model and found a sensitivity of 68.6% (95% CI, 35.0–90.9%) and a specificity of 95.2% (95% CI, 84.4–99.2%). In addition, we pooled the results from just the prospective CLIA and FDA studies using a random-effects model and found the sensitivity to be 95.2% (95% CI, 83.6–99.7%) and the specificity to be 96.8% (95% CI, 87.1–99.8%).

Most (4/5) of the laboratory-based studies were from the FDA clearance application and all used sera. The pooled sensitivity from the laboratory evaluations was 98.5% (95% CI, 92.1–100%) and the pooled specificity was 95.9% (95% CI, 81.5–100.0%) (Table 3). The laboratory study identified in the literature had the lowest sensitivity compared with the FDA laboratory evaluations.

Of the prospective studies, 4 had reference testing algorithms that included or were stratified by nontreponemal test results (Table 4). Each study used rapid plasma reagin (RPR), a nontreponemal test, in their diagnostic algorithm: Matthias et al [20] and Nakku-Joloba et al [21] had data available on RPR reactivity such that we were able to stratify the results to define positivity as those both RPR and treponemal reference test reactive and nonreactive as those both RPR and treponemal reference test nonreactive in Table 4. Fakile et al [23] also defined positivity as both RPR and treponemal reference test reactive and defined negative as those RPR and treponemal reference test nonreactive, RPR reactive and treponemal reference as nonreactive, or RPR nonreactive and treponemal reference test reactive. The CLIA waiver study used an antitreponemal chemiluminescent immunoassay (CIA) as a screening test and confirmed it with RPR. If the RPR was nonreactive, the *T. pallidum* particle agglutination assay was used as the tiebreaker [16]. With those reference algorithms, sensitivity improved to a pooled sensitivity of 97.0% (95% CI, 94.8–98.6%).

## Discussion

We reviewed prior publications and regulatory data to summarize the performance of the Syphilis Health Check test. We found that the Syphilis Health Check test had over 87% sensitivity and 96% specificity in prospective studies. In addition, when using nontreponemal results to inform infection status, the Syphilis Health Check had even higher sensitivity (97%), which is clinically important given that those who require treatment may be those who have both reactive treponemal and nontreponemal results. By combining data from several studies, the precision around sensitivity and specificity estimates increased. Additionally, we showed how the test performed in multiple settings across different specimen types. The sensitivity of the test tended to be much higher in the FDA trial studies compared with the studies identified in the literature, where the pooled sensitivity was 68.6%. The FDA trials included rigorous training and oversight while the studies described in the literature did not include methods of quality monitoring. Programs supporting ongoing quality control and quality assurance should be implemented where these rapid tests are used. In addition, the FDA prospective studies used sera for testing on the Syphilis Health Check while the other prospective studies used whole-blood specimens. This difference may have also contributed to the higher sensitivity observed in most of the FDA trials.

The Syphilis Health Check rapid test is currently the only rapid point-of-care whole-blood test for syphilis that has FDA clearance. However, many other manufacturers have commercially available, rapid point-of-care tests for syphilis, some of which have undergone regulatory approval processes in settings outside the United States. For example, the World Health Organization (WHO) has a prequalification of an in vitro diagnostics process that consists of review of products’ safety, performance, design, and manufacture. The SD Bioline HIV/Syphilis Duo (Alere, Waltham, MA) is an example of a rapid test that includes treponemal antibody detection that is WHO prequalified. Other multiplex rapid tests have been CE marked, an indication that the test meets European Union standards, including the Multiplo TP/HIV (Medmira, Halifax, Nova Scotia, Canada), DPP Syphilis Screen and Confirm assay (Chembio Diagnostics, Medford, NY), and INSTI HIV/Syphilis Multiplex test (BioLytical Laboratories Inc, Richmond, Canada) in addition to single rapid syphilis tests such as the SD Bioline Syphilis 3.0 (Alere).

The results of the pooled analysis suggest that the Syphilis Health Check had good performance for the detection of treponemal antibody, although further studies are warranted to understand why the test performed better in the studies for FDA submission than in other clinical settings. The 4 prospective studies included in our meta-analysis that were not for FDA submission had a low sensitivity (68.6%). The summary findings confirm that treponemal antibody may be detected outside of traditional laboratory settings. Future studies should stratify by stage of syphilis to better understand how the test performs at each stage. In addition, further research is needed to understand if the test performs well for immunocompromised patients and those with coinfections. One of the studies we included in our analysis included samples from pregnant women; however, those used stored sera, thus future research may be warranted that includes prospective clinical testing among pregnant women. Use of rapid point-of-care treponemal tests may facilitate the timely detection, treatment, and ultimately the enhanced control of syphilis. Differences in findings between FDA submission studies and studies conducted by other investigators point to the need for ongoing quality-assurance programs including training, competency assessments, quality-control measures, and proficiency testing with implementation of these tests. Those aspects are key to a successful testing program and should be implemented whenever utilizing a point-of-care assay.

## Notes

***Acknowledgments.*** The authors thank Dr Cheng Chen for his work conducting and coordinating the literature review and Dr Samantha Katz for her assistance in working with Centers for Disease Control and Prevention library for literature searches.

***Financial support*.** This work was supported by the National Institutes of Health (grant numbers K01 AI136725 and T32 AI007384; to C. C. B.); the UCLA Center for AIDS Research at the National Institutes of Health (grant number P30 AI028697; to J. D. K.); and the UCLA Center for HIV Identification, Prevention, and Treatment Services at the National Institutes of Health (grant number P30 MH058107; to J. D. K.).

***Supplement sponsorship.*** This article appears as part of the supplement National Coalition of STD Directors, sponsored by the CDC.

***Potential conflicts of interest.*** The authors: No reported conflicts of interest. All authors have submitted the ICMJE Form for Disclosure of Potential Conflicts of Interest.
